# Prevalence of common hemoglobin variants in an afro-descendent Ecuadorian population

**DOI:** 10.1186/1756-0500-6-132

**Published:** 2013-04-04

**Authors:** Yamila Domínguez, Camilo Zurita, Diego Calvopiña, Jacqueline Villacís, Marcelo Mora

**Affiliations:** 1 Escuela Politécnica de Ejército, Av. Gral. Rumiñahui s/n, Sangolquí, Ecuador; 2 Catedra de Inmunología, Facultad de Ciencias Médicas, Universidad Central del Ecuador, Iquique s/n, Quito, Ecuador; 3 Centro Internacional de Investigaciones en la Salud-Ecuador, Av. Prensa N49-221 y Manuel Valdivieso, Quito, Ecuador

**Keywords:** Hemoglobin, Hemoglobinopathies, Capillary electrophoresis, Ecuador, Afro-descendants, Genetic disorders, Sickle cell

## Abstract

**Background:**

Hemoglobinopathies are among the most studied and frequent pathologies. These genetic disorders are considered a very important health care threat in many tropical countries. Ecuador is a tropical Latin-American country with an important presence of afro-descendants (7.2%). Afro-descendants are among the ethnic groups with higher frequency of hemoglobinopathies reported. Ambuqui is a region within the Imbabura province with an important presence of afro-descendants (>50%). The present study analyzed the frequency of the most common hemoglobin variants in an asymptomatic afro-descendent population using capillary electrophoresis.

**Findings:**

From 114 individuals, 25 (22%) reported a hemoglobin variant. All individuals that presented hemoglobin variants were heterozygotes (asymptomatic). Hemoglobin S (sickle cell trait) was the most frequent variant found (14%), followed by hemoglobin E (4.4%), Fetal (2.6%) and C (1%).

**Conclusion:**

Prevalence of hemoglobin S was consistent with populations from other countries, but it was lower than other Ecuadorian afro-descendent populations. Frequency of hemoglobin C was lower than other afro-descendent populations. This data suggests the possibility of gene flow from Native American individuals to the Ambuqui population there by lowering the frequency of their hemoglobin variants compared with other afro-descendant populations. Evaluating the frequency of hemoglobinopathies in Ecuadorian populations is essential. Despite the high frequency of these disorders, very few health care facilities implement hemoglobinopathies tests as a routine practice.

## Background

Hemoglobinopathies are a group of genetic disorders that involve a structural change in one of the subunits of the hemoglobin. These genetic disorders represent an important health care threat in tropical low income countries due to the high prevalence of hemoglobin variants in these areas. It has been estimated that between 300.000 and 400.000 babies are born with hemoglobin disorders each year(most of them in low income countries) [1].

From the several hemoglobin variants that have been described, hemoglobin S (*Hb S*), C (*Hb C*), E (*Hb E*), beta and alpha-thalassemia have been some of the most common hemoglobin variants found in Latin American Countries. Hemoglobin S, (or sickle cell trait) has been one of the most studied hemoglobin variants. This trait is responsible for the sickle cell disease, an autosomic recessive disease caused by a point mutation in the beta chain of hemoglobin. This mutation alters the structure of hemoglobin protein which can produce several complications due to a change of shape and elasticity of red blood cells [2]. However, asymptomatic heterozygote carriers (*Hb AS*) of hemoglobin S have been implicated in resistance mechanisms against malaria parasite infection. This disorder has been reported mainly in Sub-Saharan Africa, Mediterranean region, Middle East and India [1]. In Latin America, *Hb S* has been reported as one of the most common variants found among afro-descendent populations [3,4].

Other hemoglobin variants frequently detected are hemoglobin C (*Hb C*) and hemoglobin E (*Hb E*). These variants have a lysine residue instead of a glutamic acid at the 6^th^ (*Hb C*) and 26^th^(*Hb E*) positions of the B-globin chain. Hemoglobin C is frequently found in North-East African populations while hemoglobin E is more frequent in southeast Asia where malaria is endemic [1].

Thalassemias are a group of disorders that typically involve a deletion or deficient synthesis of the alpha (alpha-thalassemia) or beta (beta-thalassemia) globin chains [5]. These alterations of the synthesis of the globin chains are usually caused by deletion or point mutation on one or more genes. Clinical severity of this pathology is influenced by the amount of genes that present changes affecting the structure of the globin chain. Alpha-thalassemia occur mainly in Africa, Asia and the Mediterranean basin while beta-thalassemia are found mostly in Asia and the Mediterranean basin [6]. Alpha and beta-thalassemia have been detected in low frequencies in Uruguayan afro-descendent population as well as populations from Venezuela with different ethnic origins [3,6].

Ecuador historically has received the influx of several ethnic groups (plus several native ethnic groups). The origins and geographical distribution of afro-descendent groups in Ecuador has been slightly different to the patterns observed in other Latin American countries. Afro-descendent populations in Ecuador have a discrete distribution, where a big percentage of this ethnic group occurs in a relatively small territory (Esmeraldas and San Lorenzo within Esmeraldas province; Ambuqui and Salinas within Imbabura province) [7]. First reports of Afro-descendent populations in Ecuador were in 1553 when a shipwreck forced several African individuals to land in coastal Ecuador [8]. This population later intermixed with Native American populations. Additionally, during the seventeenth century, an important influx of afro-descendants migrated from Colombia to the Esmeraldas province [8].

Since then, several other migratory patterns have happened in Ecuadorian populations. In spite of the fact that afro-descendant populations are condensed in some finite locations, only a few studies have analyzed the frequencies of hemoglobinopathies within these locations. One of those studies [9] focused on several populations from Esmeraldas province; while another studied hemoglobinopathies from Esmeraldas and Valle del Chota populations (Imbabura province) [10]. Ambuqui is a region within Imbabura province that groups several towns (*e.g. *Chota, Carpuela, Juncal) of which approximately 54% are afro-descent (among the highest percentage in the province along with Salinas that has 57%) [7]. Considering this high frequency of afro-descendant individuals in this population, it is important to determine the frequency and diversity of hemoglobin variants in Ambuqui. This information is essential not only for national health care- purposes, but because it also establishes a point of reference for comparison of hemoglobin variants in afro-descendent populations from other Latin American countries.

Considering that Afro-descendent populations have been reported to have high prevalence of hemoglobinopathies [11], the present study analyzed the frequency of hemoglobin variants in afro-descendent individuals from Ambuqui region (Imbabura).

## Methods

One hundred and fifteen blood samples from Afro-Ecuadorian individuals from the Ambuqui region were randomly collected from a total population of 5477 individuals [12]. Afro-descendent participants sampled account for the 2.1% of the total population of Ambuqui (3.1% of population above 15 years old) and an average age of 33.5 years old (between 16 and 81 years old). Both sexes were equally represented within the data (51.3% males, 48.7% females) Within the Ambuqui region, samples were taken from Carpuela, Juncal and el Chota. Participants were informed of the objective of the study and each of them signed an informed consent form. In compliance with the Helsinki Declaration, the study had the approval (on August 14^th^ of 2012) of the ethics committee of “Centro de Biomedicina de la Universidad Central del Ecuador (COBI-UCE)”.

Two to five milliliters of blood with EDTA were collected and centrifuged at 5000 r.p.m. for five minutes. Overlying plasma was removed and pellet was vortexed for 5 seconds. Samples were run under Minicap capillary electrophoresis equipment (Sebia, Italia Srl) in order to assess the hemoglobin fractions. Following manufacturer instructions, capillary electrophoresis was done in an alkaline buffer 9.4±5. Separation of molecules was done based on the electrolyte pH and electro-osmotic flow. Hemoglobin was detected at an absorbance wave length of 415 nm.

**Figure 1:**
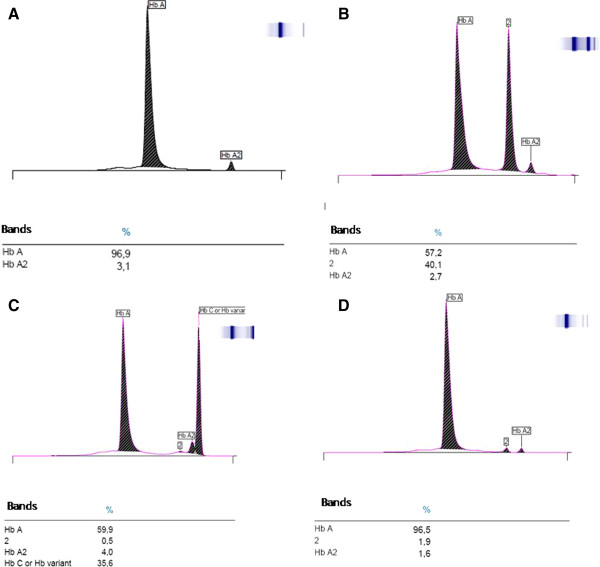
**Capillary electrophoresis pattern of hemoglobin variants done with Minicap (Sebia, Italia Srl). A**. Peak of normal Hemoglobin pattern A and A2 with their percentage are shown. **B**. Peak pattern of Heterozygote Hemoglobin S (Hb AS). **C**. Peak pattern of Heterozygote Hemoglobin C (Hb AC). (Peak 2 represents denaturated Hb C or Hb A2 variant ). **D**. Peak pattern of Heterozygote Hemoglobin E (Hb AE).

Results were interpreted according to manufacturers (Minicap, Sebia, Italia Srl) recommendations, where normal phenotype had between 96.8 – 97.8% of hemoglobin A, 2.2 – 3.2% of hemoglobin A2 and less than 0.5% of hemoglobin F (Figure 1). One extreme value of hemoglobin A (98.1%) was detected and removed from the analysis as it was considered as outlier.

## Findings

Twenty two percent of hemoglobin variants were observed in the present study (*Hb AF* 3%, *Hb AS* 14%, *Hb AE* 5%, *Hb AC* 1%, others 3%). This prevalence is consistent with the 24.3 - 25.4% reported for Afro-descendent populations located in the Cayapas and Onzoles rivers in Esmeraldas province [9,10]; but it is much higher than previously found in el Valle del Chota populations (10.5%) [10]. It is important to consider that both of these studies [9,10] were based on a much larger region comprised of several populations and thus greater variability of hemoglobin variants. For instance, Borbon and San Miguel report 12.4% and 13.1% of hemoglobin variants, while Trinidad and Santa María report 45.5% and 37.9% [9].

Results from the present study as well as from other studies in Ecuador report a higher prevalence of hemoglobinopathies in Esmeraldas and Imbabura populations than in other Latin American regions. For instance, prevalence of hemoglobin variants in a black population in Chincha province (Peru) reported 8/100 abnormal haemoglobin carriers (5 Hb *AS* and 3 Hb *AC*) [13]. Similarly, a study in several afro-descendent populations from Colombia and Costa Rica reported 7% (in 3244 samples) and 10.6% (in 621 samples) [14,15].

A study [3] based on several populations from Venezuela reported a prevalence of hemoglobin variants of 9%. However, this study was based on populations from different ethnic groups. When only Afro-descendent individuals from Venezuela were analyzed, the prevalence was 40% (798/2000), higher than other ethnic groups. Differences in frequency of hemoglobin variants have been attributed to resistance of heterozygotes (*Hb AS, Hb AC, Hb AE*) to malaria. This resistance has been used to explain differences between ethnic groups that have been historically distributed in tropical areas where there is high incidence of the malaria parasite.

**Figure 2:**
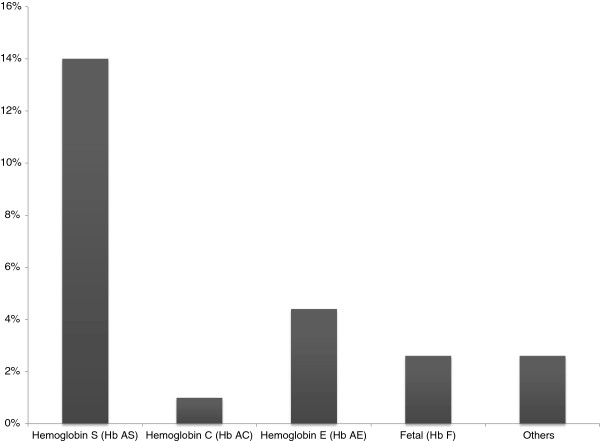
Prevalence of Hemoglobin variants observed in the afro-descendant population of Ambuqui (Imbabura).

Another study [11] in individuals from several ethnic origins in California detected a high prevalence of *Hb AS*, *Hb SS*, *Hb SC*, *HbSBthal* genotypes in black individuals. Similar results were observed in Venezuela [3] where most hemoglobin C and S trait carriers were Afro-descendants and very few were Native Americans. The afro-descendent population of the present study showed a high prevalence of *Hb S.* Fourteen percent of individual were carriers of hemoglobin S (*Hb AS*) (Figure 2). No homozygotes for sickle cell anemia were observed in the present study. Similar results were found in the afro-descendent populations from Esmeraldas were the prevalence of *Hb AS* (19.2 - 27%) [9,10]. This high prevalence contradicts the 0.5% of *Hb AS* observed an afro-descendent population in Santo Domingo (Santo Domingo de los Tsachilas province, Ecuador) [16]. There is no information on the frequency of hemoglobipathies in other Ecuadorian ethnic groups, so these results are not enough to suggest that the high prevalence of *Hb S* is restricted to afro-descendent populations. Still, it is expected that prevalence of hemoglobin variants in Native Americans and Mestizos is much lower than in Black individuals, since these two ethnic groups have shown lower frequency of hemoglobin variants than black individuals in studies from Venezuelan, Californian, and Mexican populations [3,11,17].

Frequency of hemoglobin E (4%) in the present study was higher than hemoglobin C (1%) (Figure 2).These results were unexpected as *Hb E* has a higher frequency on Southeast Asian populations, while *Hb C* has higher prevalence in North-African populations [1]. Even though hemoglobin E has been related to Asian populations, it has been found before in afro-descendent populations in Mexico, Surinam, Brazil, Spain, Jamaica and Costa Rica [18]. The Ambuqui population has a lower frequency of *Hb C* than populations from Santo Domingo de los Tsachilas (8.3%) [16]*,* Costa Rica (between 3.8% al 34.1%) [4] and populations from Venezuela (6.5%) [3].

No thalassemia’s were found in the present study. Thalassemias are more frequent in sub-Saharan Africa, the Mediterranean regions and Middle East [1]. It has been suggested that Beta-thalassemia was introduced to South America through European contact [3]. Since historically there has been low gene flow between Caucasian and Afro-descendent populations, results from the present study are expected. Additionally, afro-descendent Ecuadorian populations had some gene flow from Native American individuals. Studies indicate that Native American individuals have lower hemoglobin variants than Afro-descendants and mestizos [3]. On the other hand, alpha-thalasemia, has been reported with high prevalence in afro-descendent populations from Uruguay (15.4%) and Brazil (20 - 25%) [6,19].

Hemoglobin F is a variant, the expression of which, under normal conditions, declines after the first months of life, so after the newborn is six months old, only 5% of this hemoglobin is detected [2]. In the present study the hemoglobin F was found in a lower frequency (3%) than Esmeraldas (9.6%) [9].

## Conclusion

Ambuqui population has a relatively high frequency of *Hb AS* of 14%. This high frequency is expected in afro-descendent populations, but it is lower than the frequency observed in most populations (from 12.4% to 40.5%) from the Cayapas River, Esmeraldas [9,10]. Compared with afro-descendent populations from other Latin American countries, *Hb AS* frequency from the Ambuqui population was higher than populations from Peru (5%), Colombia- (4.65%) and Costa Rica (8.2%), Uruguay (10%), Brazil (0-13%) but lower than Venezuela (19.1%) [3,6,13-15,20]. The lower frequency reported in the Ambuqui populations compared with Esmeraldas populations could be influenced by the historical origins of afro-descendant populations in Ecuador. One hypothesis could be that populations located at Ambuqui had historically some degree of gene flow with Native American individuals, while populations at Cayapas River had an important influx of black people from Colombia. This hypothesis is supported by data that suggests that the Cayapas population genetic structure is less similar to Native American gene pool than other populations on the west part of the province [8]. Additionally, Cayapas River populations (Native-American and afro-descendant) have been reported to be under genetic and cultural isolation [21,22].

Esmeraldas populations are located, in a region with a high incidence of malaria. Resistance to the malaria parasite could still be an important factor influencing fitness of human populations in these coastal regions. Hemoglobin E had higher prevalence than *Hb C*, which is unexpected since *Hb E* has higher frequency in South East Asia.

Interestingly, patterns observed in hemoglobin variants detected in the present study and in Esmeraldas [9] differs from those observed in Santo Domingo de los Tsachilas [16]. The latter population has frequencies of hemoglobin S much lower than Esmeraldas [9] and Ambuqui, as well as, higher hemoglobin C than Ambuqui, Costa Rica [4] and Venezuela [3].

The present study found hemoglobin A, S, C and E. No thalasemias were found in the Ambuqui population. The objective of this research was to focus only on the most frequent hemoglobin variants; many other hemoglobin variants (e.g. hemoglobin M) could be analyzed in future studies. Information on hemoglobinopathies in Latin- America is still limited, considering there are more than 1000 variants of hemoglobin described so far [23]. Even though all the participants of the study who had hemoglobin variants were asymptomatic carriers, the present study showed that these variants are found in high frequencies in some populations. It will be important for future studies to assess the prevalence of hemoglobin variants in other ethnic groups. This data can then be used to assess the possibility of implementing hemoglobin tests as routine practice in hospitals in Ecuador.

## Competing interests

The authors declare that they have no competing interests.

## Authors’ contributions

YD performed the sample collection and processing, capillary electrophoresis, helped drafting the manuscript. CZ conceived of the study, and participated in its design and coordination and helped to draft the manuscript. DC helped with sample processing and capillary electrophoresis. JV helped with sample processing and capillary electrophoresis. MM wrote and edited the manuscript, helped interpret results. All authors read and approved the final manuscript.
